# Potential mechanisms of natural metabolites and botanical drugs foumulae for the treatment of non-alcoholic fatty liver disease: targeting the gut microbiota to modulate the immune system

**DOI:** 10.3389/fphar.2025.1653372

**Published:** 2025-09-25

**Authors:** Yutian Zhang, Lang Liu, Ruihao Song, Ziyi Qu, Tianlin Wang, Lei Liang, Shunhua Wang, Shuzhi Zhang, Huizhen Li, Hong Wang

**Affiliations:** ^1^ Graduate School, Tianjin University of Traditional Chinese Medicine, Tianjin, China; ^2^ College of Meteorology and Oceanography, National University of Defense Technology, Changsha, Hunan, China; ^3^ Department of Acupuncture, Qingdao Central Hospital, University of Health and Rehabilitation Sciences (Qingdao Central Hospital), Qingdao, Shandong, China; ^4^ Department of Nephrology, Shenzhen Traditional Chinese Medicine Hospital, The Fourth Clinical Medical College of Guangzhou University of Chinese Medicine, Shenzhen, Guangdong, China; ^5^ Department of Gastroenterology, The Second Affiliated Hospital of Tianjin University of Traditional Chinese Medicine, Tianjin, China; ^6^ Department of General Surgery, The Second Affiliated Hospital of Tianjin University of Traditional Chinese Medicine, Tianjin, China

**Keywords:** gut microbiota, metabolite, immune, NAFLD, NASH, botanical drug

## Abstract

Non-alcoholic fatty liver disease (NAFLD) has become the most prevalent liver disorder worldwide and is also a significant risk factor for triggering non-alcoholic steatohepatitis (NASH), hepatic fibrosis, and liver cirrhosis. Disorders in the hepatic immune system constitute one of the key drivers of NAFLD progression; thus, targeting immune dysregulation may represent an effective strategy to delay or reverse NAFLD advancement. Meanwhile, gut microbiota (GM) and its metabolites directly influence liver immune responses throughthe “Gut-Liver Axis.” Dysbiosis of the GM triggers damage to the intestinal mucosal barrier. Subsequently, substantial bacterial metabolites derived from GM can induce overactivation of the hepatic immune response, thereby driving NAFLD progression. Thus, targeted intervention in the GM-immune response axis represents an effective therapeutic approach against NAFLD advancement. Numerous current studies indicate that botanical drugs and their metabolites can counteract NAFLD progression by intervening in GM and its metabolites to regulate hepatic immune imbalance. This article reviews the roles of immune cells, GM, and their metabolites in NAFLD development, while exploring the targets and/or pathways through which botanical drugs and their metabolites modulate GM and hepatic immune responses. This aims to provide a foundation for utilizing botanical drugs as natural adjuvants to address immune dysregulation during NAFLD treatment.

## 1 Introduction

**FIGURE 1:**
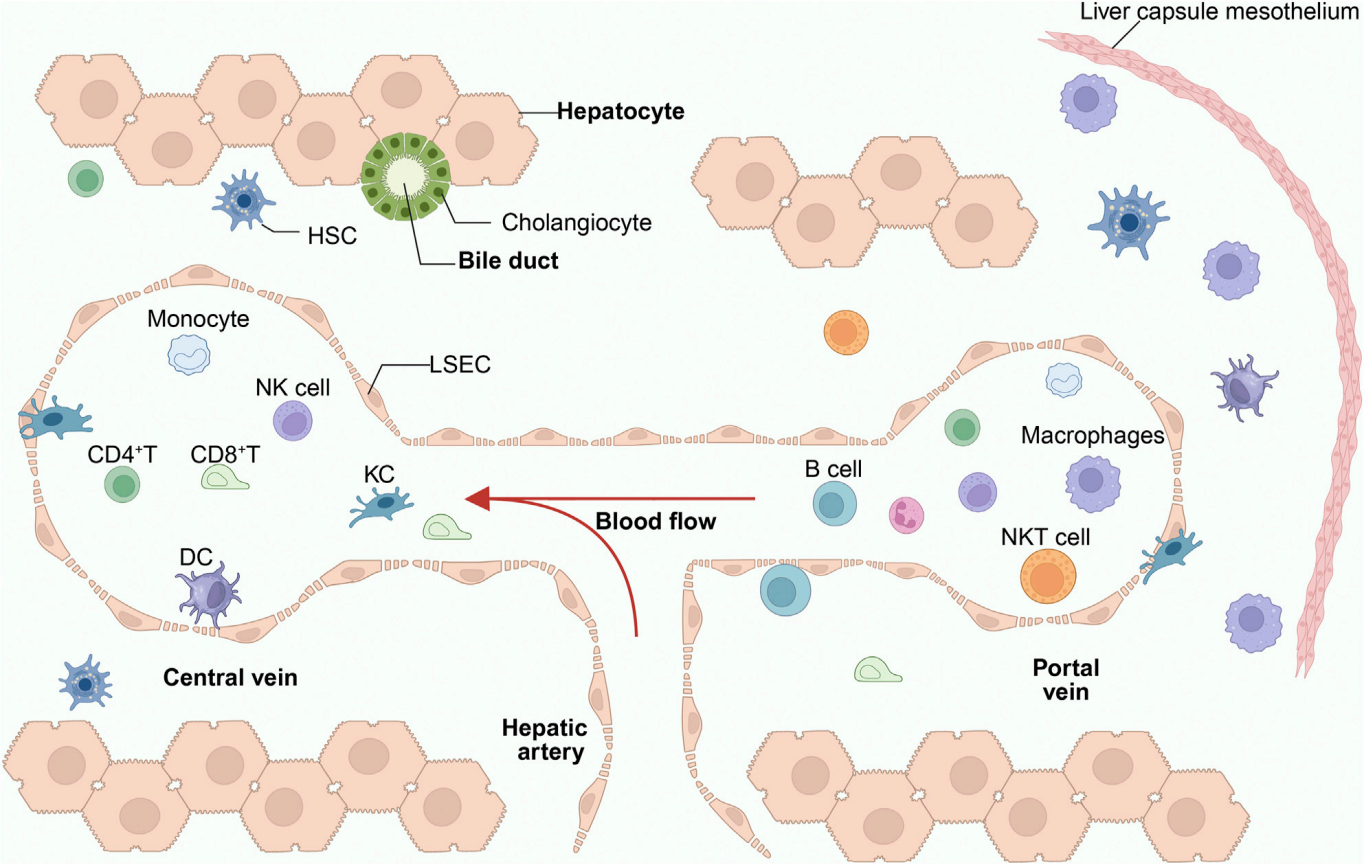
Immune status of the liver in a healthy condition. The liver lobules, composed of hepatocytes, are the primary structural units of the liver. The central vein connects to the hepatic artery and the portal vein. Bile ducts transport bile secreted by hepatocytes into the intestines. The liver contains a large number of immune cells, with the hepatic sinusoids being the primary region for their distribution. These include NK cells, LSEC, KCs, CD4^+^ T cells, DCs, and iNKT cells. Among these, KCs, iNKT cells, and CD8^+^ T cells are highly enriched in the portal vein region. KCs serve as the cornerstone of liver immunity and are closely connected to LSEC. HSCs primarily reside in the narrow Disse spaces between LSEC and hepatocytes. LCMs are a recently discovered population of macrophages in the liver, primarily distributed in the liver capsule (Glisson’s capsule). Under physiological conditions, immune cells maintain a highly organized dynamic equilibrium, simultaneously sustaining basal surveillance against pathogens while avoiding excessive inflammatory responses to harmless antigens from the intestine. KCs, Kupffer Cells; LSECs, Liver Sinusoidal Endothelial Cells; DCs, Dendritic Cells; HSCs, Hepatic Stellate Cells; NK cell, Natural Killer Cell; CD4^+^ T, CD4-Positive T Lymphocyte; CD8^+^ T, CD8-Positive T Lymphocyte; LCMs, Liver Capsule Macrophages.

NAFLD is a highly prevalent chronic progressive liver disease (Diehl and Day, 2017). It typically begins as simple hepatic steatosis, which can progress to NASH, liver fibrosis, cirrhosis, and eventually hepatocellular carcinoma (HCC). Immune responses not only determine the progression from NAFLD to NASH/liver fibrosis (Deng et al., 2022), but the retention and recruitment of immune cells within the liver also activate hepatic stellate cells (HSCs), thereby driving the development of cirrhosis and even HCC (Loomba et al., 2021). Therefore, modulating immune responses represents a promising strategy for mitigating NAFLD progression (Martínez-Chantar et al., 2021). On the other hand, there exists extensive crosstalk between the gut and the liver, which is referred to as the “gut–liver axis” (Tilg et al., 2022). The anatomical and functional connections between the gut and the liver make this axis a crucial pathway for bidirectional communication between the GM and the liver (Wang R. et al., 2021). As shown in Figure 1. A balanced GM and intestinal barrier integrity are essential not only for maintaining the homeostasis of the gut–liver axis (Schnabl, 2013), but also for ensuring hepatic immune stability in the host (Wang J. et al., 2023). Disruption of intestinal homeostasis can alter the immune status of the liver. Dysbiosis of the GM can compromise the intestinal mucosal barrier. Microbial components may enter the systemic circulation via a impaired intestinal barrier in the form of extracellular vesicles (EVs), and bind to pathogen recognition receptors (PRRs) in the liver as pathogen-associated molecular patterns (PAMPs) (Wang R. et al., 2021). This interaction leads to overactivation of immune cells, exacerbation of hepatic inflammation, and stimulation of HSCs, thereby promoting the development of liver fibrosis (Vajro et al., 2013). Additionally, metabolites derived from GM can act as damage-associated molecular patterns (DAMPs) by binding to PRRs on the surface of liver cells such as Kupffer cells (KCs), liver sinusoidal endothelial cells (LSECs), and cholangiocytes, thereby inducing hepatic immune responses and aggravating inflammatory liver injury (Wang R. et al., 2021). Thus, the GM can modulate liver immunity via the gut–liver axis, thereby influencing the progression of NAFLD.

The pathological mechanism of NAFLD is highly complex, making single-target therapies largely ineffective. To date, only Resmetirom has been approved for treating NASH patients with moderate hepatic fibrosis (F2∼F3 stages). Botanical drugs—natural resources containing multiple bioactive compounds—possess broad pharmacological actions, low toxicity, and high safety profiles, demonstrating excellent potential for treating chronic progressive diseases. Currently, botanical drugs have emerged as significant clinical agents (Hu et al., 2023), attracting considerable research attention. A growing body of studies (Che et al., 2022; Zhu et al., 2023) confirms that botanical drugs can repair damaged intestinal barriers, promote structural remodeling of GM beneficial to host health, alter the production of GM metabolites, and consequently regulate hepatic immune responses. Furthermore, botanical drugs modulate autophagy to promote apoptosis (Niazpour and Meshkani, 2025), inhibit HSC activation and hepatocyte apoptosis to counteract hepatic fibrosis (Wang et al., 2024b), and activate SIRT1 to reduce lipid accumulation and ferroptosis (Liu Y. et al., 2025), and other mechanisms to inhibit NAFLD progression. This also reveals a novel therapeutic strategy for NAFLD. Building upon this foundation, this review synthesizes current research on botanical drugs intervening in NAFLD development through the GM-liver immune axis, thereby providing theoretical support and clinical references for future botanical drug-based NAFLD interventions.

## 2 Immune cells and NAFLD

**FIGURE 2:**
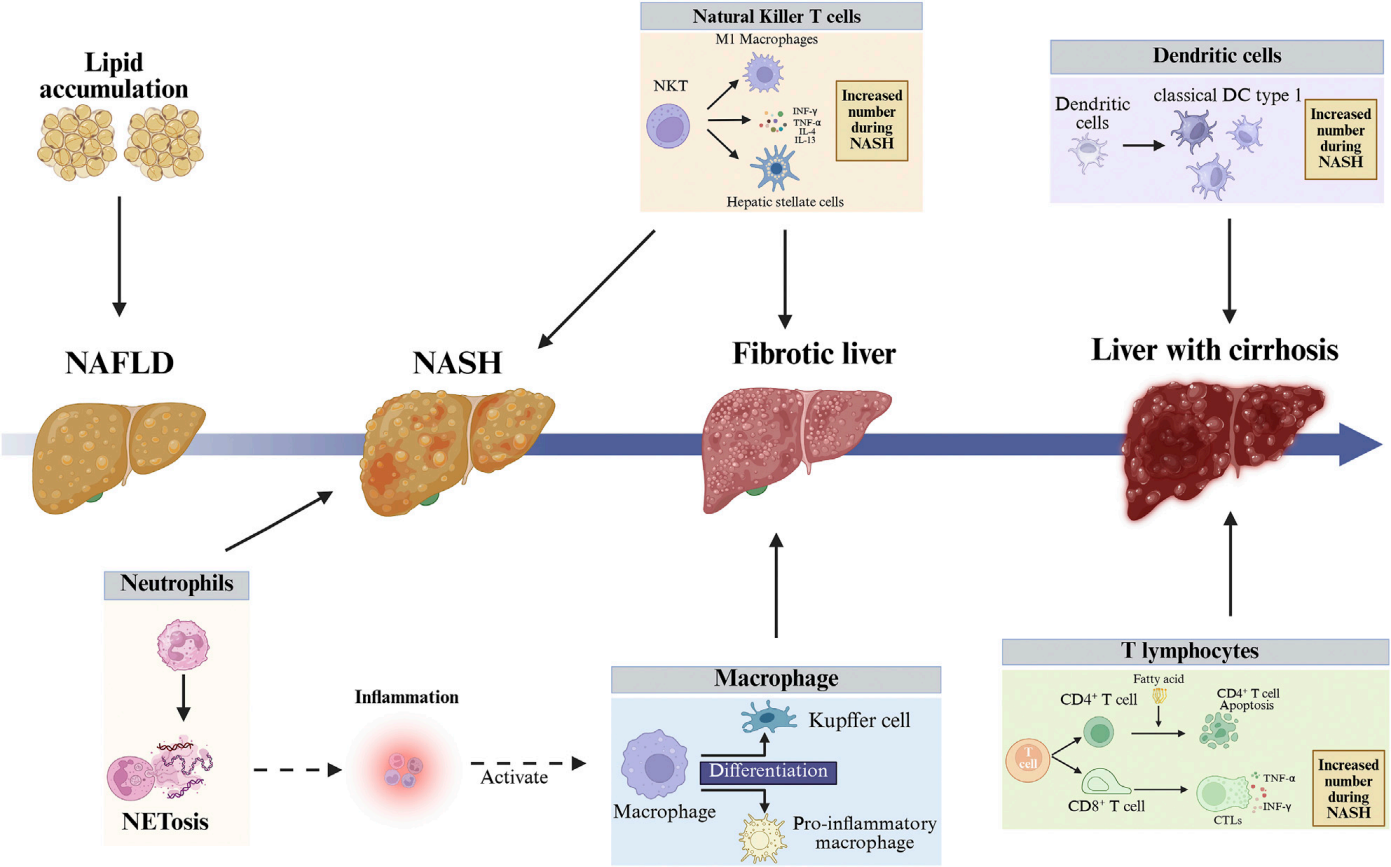
Imbalance in the liver’s immune environment can lead to liver cell death and liver fibrosis, further exacerbating liver damage. In the early stages of NASH, neutrophils become enriched, and neutrophils exacerbate liver inflammation and liver damage by secreting NETs or through NETosis. cDC1s are an important functional subpopulation of DCs, and their numbers significantly increase in the NASH stage, thereby exacerbating liver immune responses and liver damage. During the progression from NAFLD to NASH, the function of macrophages shifts from “protective” (lipid clearance) to “destructive” (inflammation-driving), with embryonic-derived macrophages differentiating into KCs and recruited monocytes differentiating into pro-inflammatory macrophages. T lymphocytes can be divided into CD4^+^ T and CD8^+^ T cells. High levels of free fatty acids in the liver and blood of NAFLD patients induce endoplasmic reticulum stress and oxidative stress in CD4^+^ T cells, ultimately leading to CD4^+^ T cell autophagy and downregulation of immune suppressive capacity. Fatty acids activate quiescent CD8^+^ T cells and drive their differentiation into CTLs. CTLs damage hepatocytes by releasing perforin, Fas ligand, and pro-inflammatory cytokines, exacerbating liver inflammation. NKT cells are activated by free fatty acids, releasing pro-fibrotic cytokines and driving macrophage M1 polarization, thereby activating HSCs. The activation of numerous immune cells disrupts liver immune balance, exacerbating liver inflammation and damage. NASH, Non-alcoholic Steatohepatitis; NAFLD, Non-alcoholic Fatty Liver Disease; NETs, Neutrophil Extracellular Traps; cDC1s, Conventional Dendritic Cells Type 1; KCs, Kupffer Cells; CD4^+^ T, CD4-Positive T Lymphocyte; CD8^+^ T, CD8-Positive T Lymphocyte; CTLs, Cytotoxic T Lymphocytes; NKT, Natural Killer T cell; HSCs, Hepatic Stellate Cells.

As one of the core organs for immune regulation, the liver harbors abundant immune cells that participate in immune responses (Peiseler et al., 2022). Under immune homeostasis, immune cells disperse throughout the liver to expel toxic substances while phagocytosing and eliminating pathogens (Bogdanos et al., 2013). Translocation of gut-derived immune signals to extraintestinal sites triggers hyperactivation of the immune system (Powell et al., 2021), promoting KCs activation in the liver and recruitment of circulatory macrophages to hepatic tissue (Golabi et al., 2019), thereby intensifying the accumulation and infiltration of inflammatory factors within hepatocytes (Nati et al., 2016). Persistent hepatic inflammation not only induces hepatocyte injury and necrosis, driving NAFLD progression to NASH, but also activates HSCs to accelerate hepatic fibrosis, cirrhosis, and HCC (Kechagias et al., 2020). Research (Huby and Gautier, 2022) has established that NAFLD progression is closely associated with macrophages, neutrophils, dendritic cells (DCs), and natural killer T lymphocytes (NKTs). As shown in Figure 2.

These immune cells synergistically interact through complex networks of cytokines and chemokines, forming an inflammatory cascade. During the progression of NAFLD from early simple fat accumulation to inflammation, fibrosis, and ultimately cirrhosis, the roles of various immune cells exhibit significant stage-dependent changes. In the early stage of fatty liver, excessive lipid deposition in hepatocytes triggers macrophages to initiate inflammation. During the hepatitis phase, macrophages serve as inflammation amplifiers, neutrophils act as executors of early liver injury, while T cells, DCs, and NKT cells participate in amplifying the inflammatory cascade. During the fibrotic stage, macrophages and NKT cells serve as pivotal pro-fibrotic drivers. In the cirrhosis phase, DCs and T cells sustain chronic immune activation while neutrophils persistently promote inflammation, collectively maintaining chronic low-grade inflammatory responses (Huby and Gautier, 2022).

### 2.1 Macrophages

Hepatic macrophages can be categorized into resident KCs (Res-KCs) and monocyte-derived macrophages (MDMs) based on their cellular origins (Guilliams and Scott, 2022). Lipotoxicity suppresses self-renewal of Res-KCs and induces their apoptosis (Tran et al., 2020; Daemen et al., 2021), thereby inducing monocyte recruitment to the liver and their differentiation into MDMs to replenish the macrophage pool (Guilliams and Scott, 2022). Res-KCs promote liver regeneration by clearing cellular debris and extracellular matrix (Crespo et al., 2023); whereas MDMs typically exhibit high expression of inflammation-related genes, exacerbating liver injury (Tran et al., 2020). Notably, TREM2 expressed in MDMs can reverse their pro-fibrotic function, exerting protective effects through facilitating the clearance of apoptotic hepatocytes and reducing inflammatory factor production (Wang X. et al., 2023). Based on inflammatory phenotypes, macrophages are classified into M1 and M2 subtypes. M1 macrophages drive fibrosis progression by secreting IL-6, TNF-α, various interleukins, and chemokines (CXCL9–11, CCL15/20) (Trinchieri, 2003; Mantovani et al., 2004; Martinez et al., 2006). Conversely, M2 macrophages exert anti-inflammatory effects by expressing TGF-β and IL-10, thereby inhibiting NAFLD progression (Hesse et al., 2001). Studies demonstrate that depleting KCs and MDMs alleviates hepatocyte steatosis, inflammation, and fibrosis, indicating their critical role in NASH pathogenesis (Bartneck et al., 2015; Cynthia and Frank, 2016). Macrophages exhibit dual regulatory functions in NAFLD (Jd and Bl, 2021), offering novel insights for targeted therapies, though challenges persist (Ginhoux et al., 2022). First, macrophage phenotype classification lacks standardization. Second, specific subtypes demonstrate functional complexity and pleiotropy. For instance, while M1 macrophages promote inflammation, they also exert anti-fibrotic effects by phagocytizing debris and secreting MMP-9 to degrade ECM (Heymann et al., 2009). Therefore, clarifying macrophage subtypes and their functions is crucial for developing precise therapeutic strategies.

### 2.2 Neutrophils

Neutrophils are among the earliest responding immune cells in hepatic inflammation (Rawat and Shrivastava, 2022). In NASH, significant neutrophil infiltration around hepatocytes not only characterizes the disease but also correlates closely with disease progression (Nati et al., 2016). The neutrophil-to-lymphocyte ratio (NLR), as a non-invasive indicator, shows positive correlation with both the NAFLD Activity Score (NAS) and fibrosis staging (Peng et al., 2018). Neutrophils drive hepatic inflammation and directly promote fibrosis through the release of Reactive Oxygen Species (ROS), proteases, Neutrophil Extracellular Traps (NETs), and inflammatory factors (Shrestha and Hong, 2023), among which NETs play a particularly prominent role (Fa et al., 2023). Neutrophils release NETs via a death mechanism known as NETosis (Brinkmann et al., 2004). NETs not only directly cause cellular damage but also induce autoantibody production through immune complex formation, triggering secondary tissue injury (Branzk et al., 2014). Studies have demonstrated that inhibiting NET formation alleviates hepatic inflammation and fibrosis in NASH mouse models while delaying their progression to liver cancer (Zhao et al., 2020). Mechanistically, NETs activate HSCs by triggering the cyclooxygenase-2/prostaglandin E2 pathway through TLR3 signaling, thereby promoting fibrogenesis (Xia et al., 2025). Neutrophil depletion also effectively ameliorates hepatic inflammation and injury in NASH mouse models (Zang et al., 2015). Furthermore, neutrophils indirectly drive NET formation via Notch signaling, exacerbating hepatocyte senescence and lipotoxicity (Xu et al., 2025). NETs can induce intrahepatic microthrombus formation, accelerating the progression from NASH to hepatic fibrosis (Tripodi et al., 2011; Du et al., 2022). This process is associated with their promotion of thrombin and fibrin generation, upregulation of tissue factor expression, and activation of coagulation factor XII (Folco et al., 2018; Shi et al., 2021). Currently, whether NETs-mediated coagulation abnormalities can serve as therapeutic targets for alleviating NASH fibrosis remains understudied and warrants further investigation.

### 2.3 Dendritic cells

DCs are specialized antigen-presenting cells that sense immune microenvironment changes, recognize pathogens, and detect inflammatory signals (Jenne and Kubes, 2013; Arrese et al., 2016). By transporting phagocytosed antigens to lymphoid organs and activating naive T cells, they bridge innate and adaptive immune responses (Wang H. et al., 2021). In the liver, DCs not only participate in inducing immune tolerance and regulating T cell responses (Bernsmeier and Albano, 2017; Tong et al., 2023), but also modulate intrahepatic homeostasis and the fibrotic process (Connolly et al., 2009).

In healthy livers, DCs are relatively sparse and exhibit limited capabilities in antigen phagocytosis and T cell stimulation. They primarily maintain tolerance to self-antigens by secreting IL-10 and IL-27 to promote regulatory T cell differentiation (Bernsmeier and Albano, 2017; Méndez-Sánchez et al., 2020). During NASH development, the number of hepatic DCs increases significantly, accompanied by an expansion of classical DC (cDC) progenitor cells in the bone marrow and bloodstream (Deczkowska et al., 2021). Notably, patients exhibit a substantial accumulation of XCR1-expressing cDC1s, whose abundance positively correlates with NASH severity (Deczkowska et al., 2021). Depletion of cDC1s alleviates hepatic inflammation in murine NASH models (Deczkowska et al., 2021). Activated DCs exhibit pro-inflammatory characteristics, releasing inflammatory factors and activating antigen-specific T cells, thereby exacerbating hepatic inflammation (Henning et al., 2013; Deczkowska et al., 2021). Furthermore, lipid accumulation within DCs triggers autoimmune responses, shifting DCs from a tolerogenic state to an immunogenic phenotype (Nati et al., 2016). Accumulated lipids provide precursors for eicosanoid synthesis (e.g., prostaglandins and leukotrienes) (Saka and Valdivia, 2012), while enhancing antigen-presenting function (Anderson and Roche, 2015).

Conversely, some studies report that DCs may also ameliorate NASH-related hepatic inflammation and fibrosis (Henning et al., 2013). For instance, depletion of DCs instead accelerated the progression of hepatic fibrosis (Lukacs-Kornek and Schuppan, 2013), with research indicating that DCs can reverse chemically-induced liver fibrosis through MMP-9 secretion. Specific clearance of DCs delayed fibrosis resolution (Jiao et al., 2012). Therefore, the role of DCs in NASH pathogenesis remains controversial, as DC depletion exhibits opposing effects across different studies. The precise mechanisms by which DCs intervene in NASH require further in-depth investigation.

### 2.4 T lymphocytes

T lymphocytes originate from hematopoietic multipotent stem cells in the bone marrow and can be classified into two subsets: CD4^+^ T lymphocytes and CD8^+^ T lymphocytes. Upon binding to MHC-II, CD4^+^ T lymphocytes differentiate into multiple subsets including helper T cell 1 (Th1), Th2, Th17, and regulatory T cells (Tregs); while CD8^+^ T lymphocytes are also termed cytotoxic T cells (CTLs) (Nati et al., 2016). Dysregulation of CD4^+^ T lymphocytes represents one characteristic feature in the progression of chronic liver diseases (Ficht and Iannacone, 2020). Th17 and Tregs constitute crucial CD4^+^ T lymphocyte subsets involved in NASH pathogenesis regulation. Under physiological conditions, Th17 and Tregs maintain a balanced state (Świderska et al., 2017). Th17/Treg imbalance leads to deterioration of NAFLD/NASH (Josefowicz et al., 2012; Chackelevicius et al., 2016). Inflammatory CXCR3^+^ Th17 cells accumulate in the liver, driving NAFLD progression to NASH through cytokine release and macrophage activation (Moreno-Fernandez et al., 2021). Studies indicate that the intrahepatic and peripheral Th17/Treg ratio in NAFLD patients reflects disease severity and progression risk (He et al., 2017). However, the role of Treg in NASH remains controversial, with traditional views emphasizing its anti-inflammatory function (Wachtendorf et al., 2024), yet research (Dywicki et al., 2022) conversely demonstrates that Treg supplementation exacerbates hepatic inflammation in murine NASH models and activates HSCs via the amphiregulin (Areg)-epidermal growth factor receptor (EGFR) pathway (Savage et al., 2024).

CD8^+^ T cells predominantly exert pro-inflammatory effects in NASH by secreting cytotoxic molecules such as IFN-γ, TNF-α, and perforin to induce target cell apoptosis (Wong and Pamer, 2003). NASH patients exhibit increased numbers of activated CD8^+^ T cells in both hepatic tissues and systemic circulation (Bhattacharjee et al., 2017). Notably, CXCR6^+^ CD8^+^ T cells exacerbate hepatic inflammation through an acetate-driven gut-liver axis (Dudek et al., 2021). Depletion of CD8^+^ T cells reduces the risk of NASH-associated HCC development (Pfister et al., 2021). However, some studies indicate that specific CD8^+^ tissue-resident memory T cells (CD8^+^ Trm) can alleviate fibrosis by inducing apoptosis in activated HSCs (Koda et al., 2021). Furthermore, CD8^+^ T cell function is regulated by the metabolic environment: it activates HSCs in high-fat diet (HFD) models but does not significantly alter liver injury or fibrosis levels in choline-deficient high-fat diet (CD-HFD) models (Breuer et al., 2020). Evidently, T cells and their subsets play diverse and sometimes opposite roles in NASH, which involves a complex immunoregulatory network. Developing strategies targeting specific T cell subsets to inhibit NAFLD may offer novel therapeutic approaches for NASH.

### 2.5 Natural killer T lymphocytes

Natural killer T (NKT) cells are a specialized T cell subpopulation that co-express T cell receptors (TCR) and NK cell receptors (Zhang and Zhang, 2020), capable of recognizing lipid antigens (such as endogenous sphingolipids or exogenous α-galactosylceramide) presented by CD1d molecules through semi-invariant TCRs (Hung et al., 2017). NKT cells can be categorized into two main subtypes: invariant (iNKT) and diverse (dNKT), with the former typically dominating pro-inflammatory responses while the latter exhibits anti-inflammatory regulatory functions (Tang et al., 2022).

During NAFLD progression, NKT cells demonstrate distinct stage-specific and context-dependent characteristics. During the simple fatty liver stage, iNKT cells suppress macrophage M1 polarization by producing anti-inflammatory factors such as IL-4 and IL-10. When the disease progresses to NASH, the lipotoxic microenvironment (e.g., free fatty acids and oxidized lipids) induces upregulation of CD1d expression in hepatocytes, thereby activating NKT cells (Tajiri and Shimizu, 2012). Activated NKT cells explosively secrete pro-inflammatory factors including IFN-γ, TNF-α, and IL-17, while recruiting neutrophils and monocytes via chemokine pathways, thereby exacerbating hepatic inflammation. (Arrenberg et al., 2011; Maricic et al., 2015; Mathews et al., 2016). Moreover, iNKT cells can directly activate HSCs through the Hedgehog signaling pathway (Nimmerjahn et al., 2005), and promote fibrogenesis via mediators such as osteopontin (OPN) (Marrero et al., 2015). Notably, IL-4 derived from NKT cells induces GARP protein expression on HSCs, thereby activating TGF-β signaling and driving hepatic fibrosis. Traj18 gene knockout in mice leads to NKT cell deficiency, consequently suppressing GARP expression on HSCs and ultimately delaying NASH progression (Zhang et al., 2023).

NKT cell functionality is also modulated by GM: Bacteroides-derived lipid antigens regulate their activation state (Wieland Brown et al., 2013; An et al., 2014). Fecal microbiota transplantation experiments demonstrate that GM from alcoholic hepatitis patients can induce liver injury in mice, while restructuring the GM prevents alcohol-induced liver injury (Llopis et al., 2016). However, the specific mechanisms underlying microbiota–NKT cell interactions remain to be elucidated (Marrero et al., 2018). Notably, NKT cells constitute 20%–30% of lymphocytes in mouse livers, but account for less than 5% in human livers (Exley and Koziel, 2004). Therefore, extrapolating pathological significance from mouse models to humans requires particular caution. Future research should prioritize validation with human-derived samples and conduct in-depth analyses of regulatory mechanisms among different NKT cell subsets within disease microenvironments.

## 3 Crosstalk between gut microbiota and immunity in NAFLD

**FIGURE 3:**
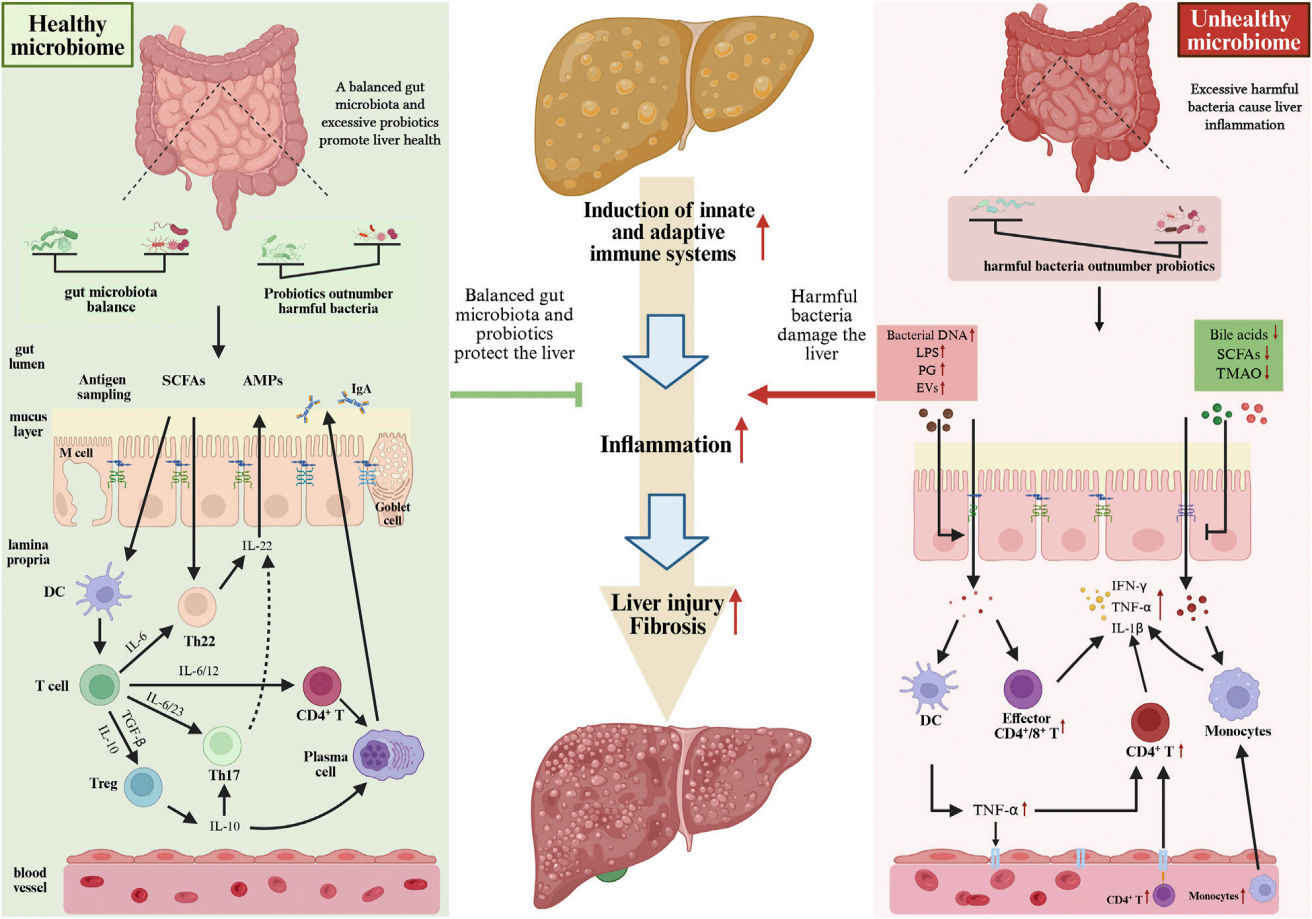
Different states of the GM can influence liver immune balance, thereby interfering with the progression of NAFLD to NASH. When the GM is in a balanced state or when the number of probiotics exceeds that of harmful bacteria, it releases various beneficial metabolites, which are crucial for maintaining liver immune balance. However, disruptions in the GM and its metabolites (where harmful bacteria outnumber beneficial bacteria) can induce liver immune imbalance, ultimately driving the progression of NAFLD to NASH. GM, gut microbiota; NAFLD, non-alcoholic fatty liver disease; NASH, non-alcoholic steatohepatitis.

GM participates in the onset and progression of NAFLD through the Gut-Liver Axis (Wu et al., 2021). GM dysbiosis and intestinal barrier damage facilitate the entry of microbial metabolites into systemic circulation, including LPS, peptidoglycan, bacterial DNA, EVs, and trimethylamine N-oxide (TMAO), which activate hepatic immunity and promote inflammation (Song and Zhang, 2022). Certain metabolites, including SCFAs, bile acids (BAs), and tryptophan metabolites, exhibit anti-inflammatory and hepatoprotective effects (Stiglund et al., 2019). Intervention targeting GM has become a crucial strategy for NAFLD treatment (Agus et al., 2021), and their interaction mechanisms with immunity offer new directions for future therapies. As shown in Figure 3.

### 3.1 Alterations in gut microbiota composition and abundance in non-alcoholic fatty liver disease

GM exhibits significant inter-individual variations, which are closely associated with geographical regions, dietary patterns, and other factors (Sarfraz et al., 2022; Procházková et al., 2024). For example, a study of populations across different regions in China revealed (Zhang J. et al., 2024) that northern residents exhibited higher abundance of *Bifidobacterium* in their GM, while southern residents showed enrichment of *Blautia* and *Lachnospiracea incertae sedis*, potentially associated with differences in dietary habits. GM directly influences the efficacy of botanical drugs, and its biotransformation capabilities are crucial for the metabolites derived from botanical drugs to exert therapeutic effects. Variations in GM composition and metabolic functions among individuals may lead to inconsistent therapeutic outcomes from identical botanical drug interventions. For instance, ginsenoside Rb1 requires transformation by specific bacterial strains into the highly active Compound K to exert anticancer effects, the absence of these specific bacteria compromises the efficacy of ginsenoside intake (Wan et al., 2017). Significant differences exist in GM composition between NAFLD patients and healthy individuals (Quesada-Vázquez et al., 2022). The pathogenesis of NAFLD is negatively correlated with the alpha-diversity of GM (Alferink et al., 2021). Multiple studies indicate that NAFLD development is associated with reduced GM diversity and alterations in specific bacterial genera: *Ruminococcaceae* and *Veillonellaceae* show positive correlation with hepatic fibrosis severity and demonstrate pro-NAFLD effects in mouse models (Lee et al., 2020).

GM structure undergoes dynamic changes across different pathological stages of NAFLD. During NAFLD progression, Gram-negative bacteria (particularly LPS-producing genera such as *Escherichia*, *Prevotella*) increase, while SCFA-producing Gram-positive bacteria (e.g., *Ruminococcaceae*) decrease (Li F. et al., 2021). Notably, through comparative analysis of GM between NAFLD patients and non-NAFLD individuals, researchers proposed *Phascolarctobacterium*, *Slackia*, and *D. formicigenerans* as biological signatures of NAFLD patients (Leung et al., 2022). However, the small sample size necessitates further validation of these conclusions. During the NASH stage, the abundance of *Clostridium coccoides* significantly increases, and hepatic fibrosis progression exhibits a positive correlation with *Ruminococcus* abundance (Boursier et al., 2016). Patients with hepatic fibrosis show reduced abundance of *Enterococcus faecalis* and *Faecalibacterium prausnitzii*. Butyrate produced by these bacteria plays a crucial role in maintaining intestinal barrier integrity (Kwan et al., 2022). Studies indicate that compositional characteristics of GM can differentiate between early and late stages of hepatic fibrosis (Loomba et al., 2017). Stable GM helps maintain hepatic immune tolerance and suppresses excessive inflammation, whereas GM dysbiosis can induce chronic hepatitis and promote the formation of a microenvironment conducive to NAFLD progression. Targeting GM to modulate the immune system has become a novel strategy for NAFLD treatment. For instance, *Bifidobacterium* exerts immunomodulatory effects by upregulating regulatory T cells, enhancing intestinal barrier function, and suppressing the activity of macrophages and dendritic cells (Gavzy et al., 2023). GM also provides new therapeutic targets for NAFLD-associated HCC. Modulating GM can influence immune regulatory molecules on T cell surfaces, thereby enhancing the efficacy of immune checkpoint inhibitors (e.g., anti-PD-1/PD-L1 and anti-CTLA-4 therapies) in HCC treatment (Huang M. et al., 2025). Although the mechanisms linking GM with NAFLD remain incompletely elucidated, GM-targeted intervention strategies undoubtedly hold broad application prospects.

### 3.2 GM-derived metabolites

#### 3.2.1 LPS

LPS plays a critical role in the onset and progression of NAFLD (Carpino et al., 2020; Ji et al., 2020). Following intestinal barrier damage, LPS translocates into the portal circulation. By binding to Toll-like receptor 4 (TLR4) on HSCs and KCs, it activates the NF-κB signaling pathway, promoting the expression of inflammatory factors and fibrogenic factors, thereby exacerbating hepatic inflammation and fibrosis (Reid et al., 2016). Beyond TLR4, LPS can also bind to lipopolysaccharide-binding protein (LBP), facilitating the formation of the CD14-TLR4 complex and further enhancing inflammatory responses (Csak et al., 2011). Studies demonstrate that blocking the LPS-TLR4 signaling pathway or reducing plasma LPS levels with polymyxin B significantly alleviates liver injury and steatosis in mice (Pappo et al., 1992; Xu et al., 2023). LPS also accelerates hepatic fibrosis progression by upregulating TGF-β expression, activating the small mother against decapentaplegic (Smad) pathway, and promoting transcription of type I and III collagen (Zhong et al., 2022). For instance, *Escherichia* coli-derived LPS activates macrophages via the TLR4 pathway, exacerbating liver injury, whereas inhibition of this pathway markedly mitigates hepatic lesions (Carpino et al., 2020). On the other hand, LPS also participates in immunomodulatory processes and can induce endotoxin tolerance. Through the LPS/TLR4 pathway, it promotes the expansion of monocyte-derived myeloid-derived suppressor cells (mMDSCs) in the liver and downregulates T cell populations, thereby modulating local immune responses (Schneider et al., 2022). LPS promotes CD14^+^ CD8^+^ T cells to secrete protective cytokines such as IL-6 and IL-33, and influences immune cell chemotaxis (Pallett et al., 2023). These effects are closely associated with decreased TLR4 and IRAK expression, along with altered p65/p50 ratios in NF-κB (Fan and Cook, 2004).

#### 3.2.2 Peptidoglycan

Peptidoglycan (PG), derived from gut bacteria in the host intestinal tract, constitutes the core structural component of bacterial cell walls (Meroueh et al., 2006). The diversity of GM gives rise to various types of PG (Krueger et al., 1982). As microbe-associated molecular patterns (MAMPs), PG can activate pattern recognition receptors TLR2, NOD1, and NOD2, thereby eliciting immune responses (Wagner and Cresswell, 2012; Juárez-Verdayes et al., 2013). NOD1 recognizes iE-DAP fragments while NOD2 identifies MDP fragments; both receptors activate NF-κB/MAPK signaling pathways, promote hepatic inflammation, and contribute to NAFLD progression (Travassos et al., 2004; Al Nabhani et al., 2017; Keestra-Gounder and Tsolis, 2017). In immune regulation, NOD1 perceives nutritional signals and drives neutrophil migration toward the liver, exacerbating inflammatory reactions (Dharancy et al., 2010; Meli et al., 2014). Notably, during advanced NAFLD stages, NOD1 promotes OX40L expression through metabolic reprogramming, upregulates CD8^+^ T cell activity, and thereby enhances immune responses to combat HCC (Zhang F. et al., 2024). In contrast, NOD2 primarily maintains GM balance and epithelial barrier homeostasis (Balasubramanian and Gao, 2017). Its activation stimulates Paneth cells to produce antimicrobial peptides (e.g., α-defensins) and enhances mucus secretion by goblet cells, thereby restricting bacterial translocation (Tan et al., 2015). Furthermore, NOD2 participates in host defense by modulating the MDP–NF-κB axis while moderately suppressing excessive TLR2 activation, thus alleviating intestinal inflammation (De Bruyn and Vermeire, 2017), this mechanism has been explored for therapeutic application in Crohn’s disease (Al Nabhani et al., 2020). Metabolically, NOD1 activation promotes metabolic inflammation and insulin resistance, whereas NOD2 exhibits anti-inflammatory and metabolic protective effects. This process requires the involvement of Receptor-Interacting Serine/Threonine-Protein Kinase 2 (RIPK2) (Cavallari et al., 2020). Therefore, targeting the PG–NOD1/NOD2 signaling pathways may offer novel therapeutic strategies for NAFLD.

#### 3.2.3 Bacterial DNA

As PAMPs, bacterial DNA enters host endosomes through various endocytic pathways. Within endosomes, TLR9 recognizes bacterial DNA-derived CpG oligonucleotides, initiating the MAPK/NF-κB pathway and triggering inflammatory factor secretion (Gomes et al., 2016; Mridha et al., 2017). Moreover, TLR9 signaling can interfere with IRF7 phosphorylation through the IKKα–LC3 pathway, thereby inducing the production of Type I interferon (Hayashi et al., 2018). Additionally, the cyclic GMP–AMP synthase (cGAS)–stimulator of interferon genes (STING) pathway can also recognize bacterial DNA (Zhang and Zhang, 2025). Bacterial DNA triggers dsDNA formation, which then activates cGAS by forming a complex. This activation generates the second messenger cGAMP, which binds to STING to activate TBK1. TBK1 phosphorylates both STING and the IRF3 transcription factor. Phosphorylated IRF3 induces Type I interferon synthesis, while STING accelerates NF-κB activation through IκBα phosphorylation (Zhang and Zhang, 2025). This collectively indicates that bacterial DNA can activate innate immune responses through multiple signaling pathways, playing a significant driving role in the onset and progression of NAFLD.

#### 3.2.4 Extracellular vesicles

GM-derived EVs are bilayer membrane-structured nanoparticles released by bacteria, carrying substantial amounts of toxic microbial molecules. These particles can traverse the compromised intestinal barrier into circulation, target the liver, and participate in NAFLD pathogenesis by activating immune-inflammatory responses (Yáñez-Mó et al., 2015). Components such as LPS carried by EVs can enter the cytoplasm via the TLR4–TRIF–GBP3 pathway, activate Caspase-11, and induce inflammation through receptors including TLR2 and NOD1/2 (Bielig et al., 2011; Gu et al., 2019). Furthermore, EVs can promote hepatic inflammation and fibrosis through the TLR4 and NLRP3–GSDMD signaling pathways (Dorner et al., 2024). EVs derived from feces of NASH patients (NASH-fEVs) disrupt intestinal barrier integrity, increase permeability, and activate HSCs via the TLR/LPS pathway, thereby upregulating fibrosis-related protein expression (Fizanne et al., 2023). Notably, the impact of EVs on the liver is context-dependent: for instance, miRNA (miR-129-2-3p) derived from *Fusobacterium nucleatum* can exacerbate intestinal inflammation by promoting cellular senescence (Wei et al., 2023). Conversely, remodeling GM using the PPARα inhibitor GW6471—which increases the abundance of probiotics (e.g., *Bacteroides*) while reducing harmful bacterial populations—alleviates hepatic lipid accumulation, ferroptosis, and oxidative stress, thereby improving NAFLD (Yang X. et al., 2025). In summary, EVs exhibit dual roles in NAFLD, where their specific effects are contingent upon both their microbial origin and the host microenvironment. Further investigation into the immune-regulatory mechanisms of active components within EVs will help elucidate their pathological significance and therapeutic potential in NAFLD.

#### 3.2.5 Indole and its derivatives

Intestinal tract commensal bacteria metabolize tryptophan (Trp) into various indole derivatives, including indole-3-acetic acid (IAA), indole-3-propionic acid (IPA), indole-3-aldehyde (IAld), indole-3-lactic acid (ILA), and tryptamine (Su et al., 2022). Different bacteria possess distinct tryptophan enzymes that produce specific indole derivatives (Dodd et al., 2017). Indole derivatives accumulate in the intestinal tract and activate the Aryl Hydrocarbon Receptor (AhR) on innate lymphoid cells, thereby promoting goblet cell differentiation and mucus secretion (Powell et al., 2020), and induce tight junction protein expression (Shimada et al., 2013), enhancing the integrity and functionality of the intestinal barrier. On the other hand, AhR is widely expressed in various immune cells, such as DCs, T cells, and lymphocytes (Nguyen et al., 2010; Rothhammer and Quintana, 2019), mediating the regulation of the immune system by indole derivatives, including promoting Treg differentiation (Goettel et al., 2016), inducing T cell apoptosis (Landfried et al., 2011), and suppressing the inflammatory activity of Th17 cells (Rothhammer and Quintana, 2019), thereby alleviating hepatic inflammation. Furthermore, indole derivatives (e.g., IAA and IPA) produced by specific strains (e.g., probiotics) inhibit the NF-κB pathway, reduce levels of pro-inflammatory factors (IL-8), and promote the release of anti-inflammatory factors (IL-10) (Bansal et al., 2010). Clinical studies have revealed decreased levels of IPA and IAA in the feces of NAFLD patients. Increasing the abundance of *Bifidobacterium bifidum*—the primary source of these metabolites—significantly ameliorates hepatic steatosis and inflammation in mice (Min et al., 2024). Furthermore, indole derivatives promote the proliferation of intestinal crypt epithelial-tubular cells in mice, which plays a critical role in maintaining intestinal immune homeostasis (Powell et al., 2020).

#### 3.2.6 Bile acids

BAs are important signaling molecules generated through enzymatic conversion of cholesterol in the liver. By activating specific receptors, they regulate hepatic lipid metabolism and suppress the transcription of inflammatory factors, thereby influencing NAFLD progression. Among these, Farnesoid X Receptor (FXR) and Takeda G-protein Receptor 5 (TGR5) represent two critical BAs receptors (Chávez-Talavera et al., 2017). FXR plays a pivotal role in anti-inflammatory and immunomodulatory processes, BAs activate FXR to suppress NF-κB signaling, thereby reducing its induction of inflammatory mediators such as IFNγ and COX-2 (Wang et al., 2008). This downregulates monocyte chemoattractant protein-1 (MCP-1) expression, diminishing macrophage infiltration into the liver (Li et al., 2015). Simultaneously, FXR activation restores the intestinal vascular barrier by triggering the endothelial Wnt/β-catenin pathway, which blocks bacterial translocation and alleviates hepatic inflammation (Mouries et al., 2019). FXR signaling also acts on macrophages, NK cells, and DCs, restricting the production of pro-inflammatory factors and suppressing inflammasome activation (Sun et al., 2021; Fiorucci et al., 2022). Since TGR5 is widely distributed in cell types including HSCs, LSECs, and macrophages (Keitel et al., 2007). TGR5 activated by BAs promotes macrophage M2 polarization (Shao et al., 2022), inhibits the TLR4–NF-κB pathway (Biagioli et al., 2017; Hosseinkhani et al., 2021), suppresses NLRP3 inflammasome activation and IL-1β secretion through the cAMP–PKA signaling axis (Pols et al., 2011; Guo C. et al., 2016), and induces endothelial nitric oxide synthase (eNOS) expression to exert anti-inflammatory effects (Keitel et al., 2007). Additionally, TGR5 promotes the release of glucagon-like peptide-1 (GLP-1) in the intestinal tract, thereby improving insulin sensitivity and lipid metabolism (Thomas et al., 2009). Notably, BAs with different structures exhibit distinct effects, for example, 12α-hydroxylated BAs (12α-OH BAs) paradoxically promote HSCs proliferation by binding to TGR5. Simultaneously, they upregulate hepatic fibrosis-related proteins (α-SMA, TGF-ß, COL I, PDGF) and exacerbate fibrosis progression through activation of ERK1/2 and p38 MAPK signaling pathways (Xie et al., 2021).

#### 3.2.7 Short-Chain fatty acids

SCFAs modulate immune and metabolic responses through multiple pathways, thereby influencing the progression of NAFLD. SCFAs promote proliferation of intestinal epithelial cells, enhance expression of tight junction proteins (e.g., ZO-1, occludin, claudin-1, and claudin-2), and activate hypoxia-inducible factor (HIF) to maintain intestinal barrier integrity (Nicolas and Chang, 2019). Butyrate also induces expression of the antimicrobial peptide β-defensin-1, reducing levels of LPS-carrying bacteria and LPS (Beisner et al., 2021). Furthermore, it inhibits the increase in intestinal permeability mediated through the TLR4/myeloid differentiation factor (MyD88) signaling pathway (Nighot et al., 2017). SCFAs deficiency impairs barrier function by causing inadequate energy supply to intestinal epithelium and disrupting mucosal immune homeostasis (Matsumoto et al., 2017).

Secondly, SCFAs regulate immune cell function through G protein-coupled receptors (GPRs) and Toll-like receptors (TLRs) (Canfora et al., 2015). For instance, butyrate promotes anti-inflammatory factor IL-10 secretion and Treg differentiation via GPR109a, while suppressing release of inflammatory factors such as IL-1β, IL-6, and TNF-α through TLR4 (Feingold et al., 2014; Sam et al., 2021). SCFAs also promote Treg differentiation via epigenetic mechanisms including HDAC inhibition and enhanced histone H3 acetylation in the Foxp3 promoter region, thereby ameliorating hepatic inflammation (Furusawa et al., 2013; Park et al., 2015). Supplementation of butyrate-producing *Clostridium* butyricum B1 (CB) in the NASH mouse model reversed HFD-induced hepatic steatosis, suppressed hepatic MCP-1 and TNF-α expression, reduced pro-inflammatory factors (IFN-γ and IL-17) in both liver and intestinal tract, and increased anti-inflammatory factors (FOXP3^+^, IL-4, and IL-22). These findings were corroborated by *in vitro* experiments (Zhou et al., 2017).

Additionally, SCFAs intervene in NAFLD through energy metabolism regulation. SCFA supplementation ameliorated hepatic steatosis in mice (Shimizu et al., 2019). Acetate and propionate stimulate peptide YY (PYY) and insulin-like growth factor-1 (IGF-1) release via GPR41/43, thereby suppressing appetite and energy intake. Concurrently activates AMP-activated protein kinase (AMPK) to reduce lipid accumulation (Deng et al., 2020). Sodium butyrate regulates hepatic lipid metabolism by promoting GLP-1 secretion from intestinal L cells (Zhou et al., 2018). *Clostridium* butyricum capsules combined with rosuvastatin demonstrate superior efficacy over monotherapy in lipid regulation, anti-fibrotic effects, and liver function improvement (Zhu et al., 2022). Notably, acetate promotes liver regeneration by inducing SCD1 expression (Yin et al., 2023), providing novel directions for NAFLD treatment.

#### 3.2.8 TMAO

Circulating TMAO levels exhibit positive correlations with NAFLD incidence risk, disease severity, and all-cause mortality (Flores-Guerrero et al., 2021). TMAO promotes NAFLD progression through multiple mechanisms. TMAO elevates mitochondrial ROS levels, activates the NF-κB signaling pathway, thereby promoting NLRP3 inflammasome assembly and the release of inflammatory factors such as IL-1β. It also disrupts calcium homeostasis in pancreatic β-cells, leading to dysfunction (Kong et al., 2024). Secondly, TMAO impairs intestinal barrier function by suppressing the Wnt/β-catenin pathway and activating TLR4/MyD88/NF-κB signaling. Simultaneously, it induces LSEC dysfunction and capillarization, while promoting macrophage M1 polarization (Nian et al., 2024). Research reveals that TMAO activates the PERK signaling pathway in zebrafish liver and HepG2 cells, inducing pathological alterations including lipid accumulation, inflammation, and fibrosis (Yang et al., 2024). TMAO triggers endoplasmic reticulum stress (ERS), activates macrophages via the TLR pathway, and exacerbates inflammatory responses (Hakhamaneshi et al., 2021). Concurrently, TMAO suppresses BAs synthesis, disrupts cholesterol metabolism, aggravates intrahepatic lipid accumulation, promotes foam cell formation, and inhibits reverse cholesterol transport (RCT), thereby further compromising hepatic lipid homeostasis (Janeiro et al., 2018). Reduced TMAO synthesis can lower the risk of NAFLD onset and progression (Corbin and Zeisel, 2012).

## 4 Botanical drugs and their metabolites alleviate the progression of non-alcoholic fatty liver disease by modulating the gut microbiota-immune responses axis

Currently, botanical drugs and their metabolites are receiving increasing attention as adjuvant therapies. Through synergistic effects, they regulate multiple interconnected targets and pathways within the disease network, promoting the restoration of immune system balance and thereby reducing hepatic inflammation (Zhi et al., 2025). Numerous studies indicate they can intervene in NAFLD progression via the GM-immune responses axis.As shown in Tables 1, 2.

**TABLE 1:** The mechanism of botanical drugs in treating NAFLD by targeting GM-immune response.

Metabolites	Botanical drug	Experimental model	Regulation of GM and its metabolism	Targeting immune	Refs
Berberine (BBR)	*Coptis chinensis* Franch	C57BL/6J mice	*Bifidobacterium* ↑, *Bacteroidetes*/*Firmicutes* ↑	IL-1 ↓, IL-6 ↓, TNF-α ↓, CD14 ↓	Cao et al. (2016)
		Six-week-old SD male rats	*Faecalibacterium prausnitzii* ↓; *Bacteroides* ↑	BBR increases the expression level of occludin, improves intestinal mucosal damage, and reduces the level of inflammatory factors in serum	Li et al. (2017)
		SD male rats	Atopobiaceae ↓, Rikenellaceae ↓, Christensenellaceae ↑; Coriobacteriales ↓; Brevibacterium ↑, Papillibacter↓; gut microbiota diversity ↑	Reduce damage to the intestinal barrier, decrease the translocation of LPS from the intestines to the liver, thereby alleviating liver inflammation	Chen et al. (2023a)
		C57BL/6J mice	No specific mechanism	Inhibits JNK1 signaling and downregulates NF-KB pathway activity	Guo et al. (2016b)
Resveratrol, (RSV)	*Polygonum cuspidatum Sieb. et Zucc*	C57BL/6J mice	No specific mechanism	Activate the AMPKα-SIRT1 signaling pathway to inhibit the NF-κB inflammatory pathway	Tian et al. (2016)
		SD male rats	*Ruminococcaceae*↑, *Lachnospiraceae*↑, *Desulfovibrio*↓	Enhance the expression occludin, ZO1, and claudin-1 to improve intestinal barrier function and reduce liver inflammation	Chen et al. (2020)
		SD male rats	*Desulfovibrio*↓,*Lachnospiraceae_NK4A316_group*↓, *Alistipes*↓, *Allobaculum*↑, *Bacteroides*↑, *Blautia*↑; SCFAs ↑	Improved intestinal barrier integrity, inhibited the migration of LPS from the intestine to the liver, and alleviated low-grade inflammation in the liver	Wang et al. (2020b)
Curcumin (Cur)	*Curcuma longa* L	Male C57BL/6J mice	*Firmicutes/Bacteroidetes* ↓, *Akkermansia* ↑	Increasing the expression levels of occludin and ZO1, inhibiting the activation of the TLR4/NF-κB signaling pathway in the liver, and reducing the suppression of LPS-induced immune responses in the liver	Hong et al. (2022)
		Male C57BL/6J mice	*Firmicutes/Bacteroidetes* ↓, *Desulfovibrio* ↓ *Akkermansia* ↑, *Bacteroides* ↑, *Parabacteroides* ↑, *Alistipes* ↑, *Alloprevotella* ↑	Reduce HFD-induced hepatic steatosis and serum LPS concentration in mice, and alleviate LPS-induced hepatic inflammation	Li et al. (2021b)
		Male C57BL/6J mice	No specific mechanism	Cur effectively inhibits lipopolysaccharide and IFN-γ-induced M1 macrophage activation and reduces IL-1β and TNF-α	Tong et al. (2021)
Quercetin (QUE)	*Scutellaria baicalensis* Georgi	Male C57BL/6J mice	*Firmicutes/Bacteroidetes* ↓, *Helicobacter* ↓	Inhibits LPS synthesis, suppresses activation of the TLR4/NF-κB signaling pathway, and inhibits inflammasome activation	Porras et al. (2017)
		C57BL/6J mice	No specific mechanism	Increase the expression of SOD and GPX1 to enhance antioxidant capacity, and block the phosphorylation of IκBα and NF-κB p65 to inhibit excessive activation of the immune response	Jiang et al. (2025)
Lycium barbarum polysaccharides (LBPs)	*Lycium chinense* Mill	SD male rats	*Verrucomicrobia* ↓, *Enterococcaceae* ↓	Reduce intestinal LPS synthesis and LPS migration to the liver, and block LPS activation of KCs in the liver	Hu et al. (2020)
		SD male rats	*Butyricicoccus *↑, *Butyricimonas* ↑	Promote butyrate secretion, thereby increasing intestinal mucus and the expression of ZO-1 and occludin, and inhibiting LPS-induced liver inflammation	Gao et al. (2021)
		Human	*Bacteroides* ↑, *Bifidobacterium* ↑, *Phascollarctobacterium* ↑, *Prevotella* ↑, *Collinsella* ↑, SCFAs ↑	No specific mechanism	Ding et al. (2019)
Poria cocos Polysaccharide (PCP)	*Wolfiporia cocos* (F. A. Wolf) Ryvarden & Gilb	C57BL/6J mice	*Faecalibaculum* ↑, gut microbiota diversity ↑, LPS ↓	Inhibiting the NF-κB/CCL3/CCR1 signaling pathway to reduce immune responses in the liver	Tan et al. (2022)
		C57BL/6J mice	No specific mechanism	Improving liver cell apoptosis and repairing the intestinal barrier by inhibiting the CYP2E1/ROS/MAPKs signaling pathway to reduce liver immune response	Jiang et al. (2022)
Ginsenoside	*Panax ginseng* ** **C. A. Mey	C57BL/6J mice	*Akkermansia* ↑,*Oscillospira* ↑,*Phascolarctobacterium* ↑, *Bacteroides* ↑, *Dehalobacterium*↑, *Allobaculum *↓, *Olsenla* ↓	Increase the expression of ZO-1, Occludin, and Claudin-1 to maintain intestinal barrier function and reduce the risk of liver inflammation driven by enteric LPS.	Shi et al. (2024)
		C57BL/6J mice	No specific mechanism	Inhibiting SIRT1 and FOXO1 in the liver to interfere with NF-κB signaling and oxidative stress, thereby alleviating liver inflammation	Wu et al. (2025)
pachymic acid (Pac)	*Wolfiporia cocos* (F. A. Wolf) Ryvarden & Gilb	C57BL/6J mice	*Firmicutes*/*Bacteroidetes* ↓, *Akkermansia* ↑, *Desulfovibrio* ↓, *Streptococcus* ↓, gut microbiota diversity ↑	Inhibiting the LPS/TLR4/MYD88/NFκB signaling pathway to reduce liver inflammation. Pac downregulates the expression of FASN, SREBP1c, and SCD1 to reduce lipid synthesis, while promoting the expression of PPARα and CPT1α to enhance fatty acid oxidation, ultimately reducing liver inflammation induced by lipid accumulation	Ren et al. (2025a)

**TABLE 2:** Researches on the treatment of NAFLD by targeting the GM-immune response with botanical drugs formulae.

Botanical drugs formulae	Botanical drugs	Model	Regulation of GM and its metabolism	Targeting immune and inflammatory responses	Refs
Yinzhihuang granule (YZHG)	*Artemisia capillaris* Thunb *Gardenia jasminoides* J.Ellis;*Scutellaria baicalensis* Georgi; *Lonicera japonica* Thunb	C57BL/6J mice	*Firmicutes*↓, *Proteobacteria*↓, *Patescibacteria↑*, *Tenericutes*↑ *RuminococcaceAE_UCG-014*↑, *Lactobacillus*↑, *Desulfovibrio*↑	ZO1 *↑*, Occludin *↑*, Claudin 1 *↑*; ACC1↓, FASN ↓, CD36 ↓	Tan et al. (2023)
Xie Zhuo Tiao Zhi formula (XZTZ)	*Alisma plantago-aquatica* L;*Atractylodes macrocephala* Koidz *Wolfiporia cocos* (F. A. Wolf) Ryvarden & Gilb *Citrus × aurantium* Siebold & Zucc. ex Engl.; *Crataegus pinnatifida* Bunge *Nelumbo nucifera* Gaertn	C57BL/6J mice	*Ileibacterium valens↑, Bifidobacterium pseudolongum ↑*	Inosine inhibits the expression of focal death-associated proteins NLPR3, GSDMD, Nek7, Caspase 1 and ASC, and reduces the levels of inflammatory factors IL-1, IL-6 and TNFα	Qiu et al. (2023)
Yinchen-Gancao decoction (YG)	*Artemisia capillaris* Thunb;*Glycyrrhiza uralensis* Fisch	C57BL/6J mice	No special changes	Reduces fatty acid synthesis and uptake, increases fatty acid oxidation, and reduces inflammatory factors and chemokines to suppress hepatic inflammation and endoplasmic reticulum stress	(J et al., 2025)
Si-Wu-Tang (SWT)	*Rehmannia glutinosa *(Gaertn.) *Libosch.* ex Fisch. & C. A. Mey; *Paeonia lactiflora *Pall; *Angelica sinensis *(Oliv.) Diels *Ligusticum* chuanxiong Hort	C57BL/6J mice	*Bacteroides↑*, *Lachnoclostridium↑*;*Alistipes*↓, *Rikenellaceae*↓	Regulation of bile acid metabolism to treat liver fibrosis	Xue et al. (2021)
Lingguizhugan decoction (LGZG)	*Wolfiporia cocos* (F. A. Wolf) Ryvarden & Gilb;*Cinnamomum cassia* (L.) D. Don; *Atractylodes macrocephala* Koidz.; *Glycyrrhiza uralensis* Fisch	C57BL/6J mice	*Bacteroides↑*, *Lachnoclostridium↑*;*Alistipes*↓, *Rikenellaceae*↓	Reduction of hepatic mitochondrial damage and oxidative stress and inhibition of inflammatory factor release via STING-TBK1-NF-κB signaling pathway	Cao et al. (2022)
Yindanxinnaotong formula (YDX)	*Ginkgo biloba* Leaf; *Salvia miltiorrhiza *Bunge; *Asarum heterotropoides* F. Schmidt*; Panax notoginseng *(Burkill) F. H. Chen ex C. H. Chow; *Crataegus pinnatifida *Bunge; *Gynostemma pentaphyllum* (Thunb.) Makino; *Borneolum Syntheticum; Allium sativum *Bulb	C57BL/6J mice	*Firmicutes*/*Bacteroidetes ↑*; *Odoribacter ↑*, *Alistipes↑*, *Flavonifractor↑*, *Oscillibacter↑*, *Pseudoflavonifractor ↑*, *Desulfovibrio ↑*, *Mucispirillum↑*, *Acetatifactor ↑*, *Clostridium cluster* XIVa ↓, *Barmesiella*↓; SCFA *↑*	Protecting the intestinal barrier as well as lowering LPS levels, which can help reduce the risk of liver exposure	Huang et al. (2024)

### 4.1 Metabolites originating from botanical drugs

#### 4.1.1 Berberine

Berberine (BBR), a natural metabolite derived from *Coptis chinensis* Franch., alleviates hepatic inflammation by targeting GM. Administration of BBR (40 mg/kg) to NAFLD mouse models increased the relative abundance of *Akkermansia* and *Bacteroides* at the genus level, while decreasing the relative abundance of *Lactobacillus* and *Romboutsia* (Yang et al., 2022). BBR supplementation elevated intestinal *Bifidobacterium* abundance and *Bacteroidetes*/*Firmicutes* ratio in NASH mouse models, concurrently reducing serum concentrations of inflammatory factors including IL-1, IL-6, TNF-α, and CD14 (Cao et al., 2016). The study also found that BBR supplementation reduced the abundance of *F. prausnitzii* (Li et al., 2017). Regarding intestinal barrier function, BBR promotes the expression of tight junction proteins (ZO-1 and Occludin), increases the number of colonic glands and mucus secretion by goblet cells, reduces translocation of Gut-Derived LPS to the liver, and alleviates hepatic inflammation (Chen D. et al., 2023; Chen et al., 2023 Y.). Beyond regulating GM, BBR exerts effects through direct anti-inflammatory and anti-fibrotic mechanisms, including inhibition of JNK1 signal transduction (Guo T. et al., 2016) and downregulation of TLR4/MyD88/NF-κB pathway activity (Wang L. et al., 2020), reducing the expression and activity of neutrophil elastase (NE), upregulating α1-antitrypsin (α1-AT), and inhibiting the CXCR4/CXCL12 axis (Yang et al., 2017), as well as inducing apoptosis of HSCs and suppressing their proliferation (Eissa et al., 2018). Animal studies demonstrate that BBR supplementation (200 mg/kg/d) significantly alleviates hepatic inflammation and steatosis in HFD-induced NASH mouse models, with these effects closely linked to GM modulation and intestinal barrier repair. (Cao et al., 2016). A clinical trial involving 184 NAFLD patients further confirmed that oral BBR administration (1.5 g/d for 16 weeks) significantly reduced hepatic fat content, lipid parameters (TG, TC), and liver enzyme levels (ALT, AST), while exhibiting a favorable safety profile (Yan et al., 2015). BBR demonstrates promising therapeutic potential for NAFLD/NASH prevention and treatment through multi-target modulation of GM, enhancement of barrier function, and suppression of inflammatory signaling pathways and fibrotic progression.

#### 4.1.2 Resveratrol

Resveratrol (RSV), a natural polyphenolic metabolite primarily extracted from *Reynoutria japonica* Houtt., exhibits antioxidant, anti-apoptotic, and anti-inflammatory properties (Tian and Liu, 2020). As a natural agonist of silent information regulator 1 (SIRT1), RSV ameliorates hepatic lesions by exerting anti-inflammatory and anti-fibrotic effects through multiple pathways. In hepatocytes, RSV significantly alleviates hepatic inflammation by activating the AMPKα–SIRT1 signaling pathway to inhibit the NF-κB inflammatory pathway (Tian et al., 2016). Simultaneously, RSV induces apoptosis of activated HSCs and suppresses their activation in a dose-dependent manner via the SIRT1 and JNK signaling pathways, thereby reversing hepatic fibrosis (Zhang et al., 2022). On the other hand, RSV also exerts significant regulatory effects on immune cells. It interferes with interferon-gamma (IFN-γ)-mediated macrophage activation by inhibiting the JAK/STAT-1 pathway, consequently reducing the production of inflammatory mediators such as nitric oxide (NO), IP-10, and MIG, while downregulating the expression of inducible nitric oxide synthase (Chung et al., 2011). Additionally, RSV promotes macrophage polarization toward the M2 anti-inflammatory phenotype and upregulates IL-10 synthesis, thereby alleviating fibrosis (Yu et al., 2019; Jin et al., 2025). Furthermore, RSV inhibits NF-κB nuclear translocation and reduces inflammatory factor release by disrupting crosstalk between the TLR2/MyD88/ERK and NF-κB/NLRP3 inflammasome pathways, thereby delaying the progression of hepatic fibrosis (Lei et al., 2025). Regarding intestinal effects, RSV not only remodels GM and enhances microbial diversity but also strengthens intestinal mucosal barrier function by upregulating tight junction proteins (Occludin, ZO-1, and Claudin1). It concurrently suppresses mRNA expression of cannabinoid receptor type 1 (CB1), further consolidating its protective effects along the gut-liver axis (Chen et al., 2020).

Supplementation with RSV (50/75/100 mg/kg/d) significantly reduced hepatic steatosis and fibrosis in HFD-induced NASH rats, with the ameliorative effect demonstrating dose dependency. RSV restructured the GM composition in NASH rats. At the species level, the abundance of *Akkermansia muciniphila* increased; at the genus level, *Bacteroides* abundance increased while *Desulfovibrio* abundance decreased; at the family level, *Ruminococcaceae* and *Lachnospiraceae* abundances increased. However, these GM alterations were not observed in the low-dose RSV group (50 mg/kg/d) (Campbell et al., 2019). The study also found that RSV supplementation reduced the abundance of harmful bacteria *Desulfovibrio*, *Lachnospiraceae_NK4A316_group*, and *Alistipes* in the intestinal tract, while increasing the abundance of SCFA-producing bacteria *Allobaculum*, *Bacteroides*, and *Blautia* (Wang P. et al., 2020). An RCT involving 60 NAFLD patients demonstrated that 3 months of RSV supplementation (300 mg/d) significantly reduced serum levels of ALT, AST, TC, and TNF-α (Chen et al., 2015).

#### 4.1.3 Curcumin

Curcumin (Cur), a natural metabolite extracted from *Curcuma longa* L., exhibits multiple biological activities including antioxidant, anti-inflammatory, and antitumor effects. Cur intervenes in NAFLD progression through multiple mechanisms, including anti-inflammatory effects, regulation of lipid metabolism, improvement of insulin resistance, and modulation of fibrotic processes (Li X. et al., 2024). In recent years, its role in modulating GM has garnered increasing attention. Studies demonstrate that Cur significantly increases *Bacteroides* abundance and ameliorates hepatic lipid accumulation through microbiota-dependent BAs metabolism (He et al., 2024). In NAFLD models, Cur reverses the elevated *Firmicutes*/*Bacteroidetes* ratio and reduced *Akkermansia* abundance (Hong et al., 2022), while concurrently upregulating expression of tight junction proteins Occludin and ZO-1, suppressing TLR4/NF-κB pathway activation, and reducing LPS exposure, thereby alleviating hepatic inflammation (Hong et al., 2022). Further studies (Li S. et al., 2021; Hong et al., 2022) confirmed that Cur effectively ameliorated hepatic steatosis and reduced serum LPS levels in HFD-fed mice. This protective mechanism correlates with modulation of GM composition, specifically manifested through: decreased *Firmicutes*/*Bacteroidetes* ratio and reduced *Desulfovibrio* abundance, alongside elevated abundance of *Akkermansia* and multiple SCFA-producing genera including *Bacteroides*, *Parabacteroides*, *Alistipes*, and *Alloprevotella*. Cur supplementation (200 mg/kg/d for 16 weeks) significantly alleviated hepatic steatosis and oxidative stress in HFD-fed mice, mediated through multiple pathways (Yang J. et al., 2025). Cur enhanced the alpha-diversity of the GM. At the family taxonomic level, a significant increase in the abundance of *Coriobacteriaceae* was observed. At the genus level, the abundance of *Mailhella* and *Parabacteroides* increased, while that of *Alistipes* decreased. At the species level, the abundance of *Phocaeicola vulgatus* and *Bacteroides intestinalis* rose, whereas *Acutalibacter muris* abundance declined, thereby reducing the synthesis of enterogenic toxins. Simultaneously, Cur inhibits the JNK2/FOXO1/Bcl6 signaling axis to alleviate lipid accumulation (Yang J. et al., 2025). Moreover, Cur suppresses M1 polarization of macrophages and reduces the secretion of IL-1β and TNF-α (Tong et al., 2021). By promoting PPARα mRNA m6A methylation through inhibition of FTO protein, it activates the PPARα/CPT1α pathway to enhance fatty acid β-oxidation (Fan et al., 2025b), regulates AMPK, ChREBP, and SREBP1-c expression to ameliorate lipid metabolism (Guariglia et al., 2023), downregulates CYP2E1 and C/EBPβ, reduces ROS generation, and alleviates oxidative stress (Afrin et al., 2017). A meta-analysis encompassing 1,028 NAFLD patients also demonstrated that curcumin effectively ameliorates hepatic steatosis (Ngu et al., 2022).

#### 4.1.4 Quercetin

Quercetin (QUE), a metabolite isolated from *Scutellaria baicalensis* Georgi, intervenes in NAFLD through multiple pathways. QUE reverses HFD-induced GM dysbiosis, significantly lowering the *Firmicutes*/*Bacteroidetes* ratio and reducing the abundance of Gram-negative bacteria such as *Helicobacter*. This diminishes endotoxemia occurrence, ultimately suppressing TLR4/NF-κB signaling pathway activation and inflammasome initiation, thereby ameliorating hepatic inflammation (Porras et al., 2017). QUE supplementation also inhibits oxidative stress in hepatocytes by regulating cytochrome P450 2E1 (CYP2E1), thereby conferring hepatoprotective effects against NAFLD (Porras et al., 2017). Conversely, QUE inhibits the NF-κB p65/iNOS signaling pathway in a concentration-dependent manner and reduces serum TNF-α levels (Ying et al., 2013). It blocks phosphorylation of IκBα and NF-κB p65 while upregulating expression of superoxide dismutase (SOD) and glutathione peroxidase 1 (GPX1), thus systematically alleviating hepatic oxidative stress and excessive immune activation (Jiang et al., 2025). Furthermore, QUE can activate the antioxidant transcription factor Nrf2 and alleviate hepatic lipid accumulation, mitochondrial dysfunction, and oxidative stress through the AMPK-dependent autophagy pathway (Panchal et al., 2012; Cao et al., 2023).

The effect of QUE on ameliorating lipid metabolism is closely associated with its promotion of beneficial *A. muciniphila* proliferation. Indole-3-lactic acid (ILA), produced by this bacterium’s metabolism, upregulates CYP8B1 via the FTO/m6A/YTHDF2 pathway, driving cholesterol conversion into cholic acid (CA). The latter suppresses lipid accumulation through FXR receptor activation (Liu J. et al., 2025). A randomized controlled trial (n = 41) demonstrated the clinical value of QUE. NAFLD patients supplemented with 500 mg QUE daily for 12 weeks exhibited significant reductions in hepatic lipid content, body weight, and body mass index (BMI), with favorable safety profiles (Li N. et al., 2024).

#### 4.1.5 Lycium barbarum polysaccharides

Lycium barbarum polysaccharides (LBPs) constitute a key active metabolite in *Lycium chinense* Mill. LBPs delay NAFLD progression by modulating GM and enhancing barrier function. In NAFLD mouse models, LBPs reduce the relative abundance of *Verrucomicrobia* and *Enterococcaceae* (Gao et al., 2021), and the latter serves as the primary source of gut-derived LPS. This helps reduce the risk of LPS translocation and hepatic inflammation (Hu et al., 2020), while simultaneously increasing the abundance of *Deferribacteres* and butyrate-producing bacteria (such as *Butyricicoccus* and *Butyricimonas*) (Gao et al., 2021). Butyrate enhances the expression of intestinal mucus and tight junction proteins (ZO-1, occludin), thereby improving intestinal barrier function (Peng et al., 2007). The study (Ding et al., 2019) further confirmed that LBPs promote the proliferation of *Bacteroides*, *Bifidobacterium*, and SCFA-producing bacteria such as *Phascolarctobacterium*, *Prevotella*, and *Collinsella*. LBPs play a crucial role in inhibiting the progression of NAFLD.

Notably, the effects of LBPs on modulating GM are not solely determined by their metabolites; the structural domains of LBPs also exert influence. This structure-function relationship is particularly evident in *Bacteroidetes* and *Parabacteroides*. RG-I and its neutral sugar side chains increase the abundance of beneficial bacterial families such as *Comamonadaceae*; whereas linear homogalacturonan (HG) promotes the proliferation of potentially harmful bacterial families including *Pseudomonadaceae*, *Xanthomonadaceae*, *Caulobacteraceae*, and *Oxalobacteraceae* (Wei et al., 2025).

LBPs also stimulate putrescine secretion by murine GM, which subsequently inhibits the JAK2-STAT3 pathway via TRAF6-mediated suppression, thereby reducing Th17 cell differentiation and inflammatory factor release (Wang et al., 2025). Moreover, Th17 differentiation drives the progression from NASH to HCC, rendering the inhibition of this differentiation a crucial intervention strategy for NASH (Huang Y. et al., 2025). Supplementation with LBPs (100 mg/kg, 10 weeks) significantly alleviated liver injury, dyslipidemia, and inflammation in NASH rats. This effect was achieved by activating the AMPK/PPARα/PGC-1α pathway to promote hepatic lipid consumption (Li et al., 2022). LBP inhibited caspase-9/3 activity and TNF-α levels in CCl4-induced hepatic fibrosis mice, thereby mitigating hepatic inflammation and fibrosis (Chiang and Chao, 2018). A pre-post study also demonstrated that LBP supplementation (300 mg/day, 12 weeks) improved both GM diversity and liver function in NAFLD patients (Fan et al., 2025a).

#### 4.1.6 Poria cocos polysaccharide

Poria cocos polysaccharide (PCP) is a botanical drug-derived metabolite (Zhao et al., 2023). PCP increases the relative abundance of *Faecalibaculum*, reduces gut-derived LPS levels, thereby inhibiting the NF-κB/CCL3/CCR1 signaling pathway, alleviates hepatic inflammation, and delays NASH progression (Tan et al., 2022). Furthermore, PCP reduces hepatocyte apoptosis by suppressing the CYP2E1/ROS/MAPKs signaling pathway, and decreases the liver’s exposure risk in inflammatory environments through restoration of intestinal barrier function (Jiang et al., 2022). In both zebrafish and mouse NAFLD models, PCP demonstrated efficacy in counteracting hepatic steatosis (Ye et al., 2022). This beneficial effect stems from reducing translocation of gut-derived LPS to the liver and suppressing PARP-1-mediated pyroptosis in intestinal cells. Furthermore, PCP inhibited disease progression in methionine-choline-deficient (MCD)-induced NASH mice by downregulating expression of F4/80, CD68, IL-1β, CD11b, and CCL5 genes (Gao et al., 2023). Simultaneously, PCP alleviated hepatic steatosis in NAFLD mice by modulating glucose and lipid metabolism through upregulation of lipid transport proteins and suppression of lipid synthesis-associated proteins (Wang et al., 2022). PCP is even considered a prebiotic. Supplementation with PCP (50 mg/kg/d, for 8 weeks) can alleviate insulin resistance, lipid metabolism imbalance, and inflammation in the NASH mouse model. This effect stems from PCP enhancing the intestinal barrier, improving GM diversity, and increasing the relative abundance of probiotics including *Lactobacillus*, *Allobaculum*, and *Phascolarctobacterium*. These probiotics synthesize SCFAs, which subsequently ameliorate insulin resistance through the FGF21-PI3K/AKT signaling pathway (Liu et al., 2025b).

#### 4.1.7 Ginsenoside

Ginsenoside (Rg) is a metabolite extracted from *Panax ginseng* C. A. Mey. Rg supplementation ameliorates hepatic steatosis and reduces hepatic inflammation in HFD-induced NAFLD mouse models, with its therapeutic effects on NAFLD being closely related to modulation of the GM-immune axis (Shi et al., 2024). Studies demonstrate that Rg can restructure GM composition, and this alteration proves beneficial to host health. At the phylum level, *Bacteroidota* abundance increases while the *Firmicutes*/*Bacteroidetes* ratio decreases; at the family level, *Muribaculaceae* abundance elevates; At the genus level, the relative abundances of *Akkermansia*, *Parabacteroides*, *Lachnospiraceae_NK4A136_group*, *Oscillospira*, *Phascolarctobacterium*, *Bacteroides*, and *Dehalobacterium* increased, while the relative abundances of *Allobaculum* and *Olsenella* decreased (Liang et al., 2021; Shi et al., 2024). Concurrently, Rg enhances intestinal barrier integrity by upregulating the expression of tight junction proteins (including ZO-1, Occludin, and Claudin-1), thereby reducing the translocation of Gut-Derived LPS. Furthermore, Rg reduces LPS levels and inhibits the TLR4/NF-κB signaling pathway, consequently decreasing the production of pro-inflammatory factors and suppressing macrophage activation and inflammatory cell infiltration in the liver, ultimately alleviating hepatic inflammation (Liang et al., 2021).

Beyond the intestinal tract, Rg delays NAFLD progression by multi-target regulation of lipid metabolism. Regarding lipid metabolism, Rg reduces lipid uptake through suppression of CD36 expression (Wu et al., 2025) while activating the hepatic LKB1/AMPK/mTOR signaling pathway (Shi et al., 2024). This dual action cooperatively downregulates key lipid synthesis genes (e.g., SREBP-1c, FAS, ACC) and upregulates the fatty acid oxidation gene CPT-1a, thereby significantly ameliorating hepatic lipid accumulation (Liang et al., 2021; Shi et al., 2024). Furthermore, activated AMPK exerts hepatoprotective effects through metabolic and anti-inflammatory mechanisms: it inhibits mTORC1 and SREBP-1c to reduce lipid synthesis, while suppressing NF-κB signaling and oxidative stress via the sirtuin 1/FOXO1 pathway (Wu et al., 2025). Notably, lipid overload can induce ferroptosis, thereby driving the progression of NAFLD (Chen et al., 2022). Rg effectively enhances antioxidant capacity by activating the Keap1/Nrf2 signaling pathway and preserving mitochondrial structural and functional integrity, consequently inhibiting ferroptosis (Liu et al., 2025c). Significantly, the anti-ferroptosis effect of Rg markedly diminished following antibiotic intervention, indicating its dependence on GM involvement. This demonstrates that Rg primarily suppresses NAFLD development through multiple pathways by modulating the “GM–immune inflammation” axis while synergistically improving lipid metabolism and antioxidant pathways.

#### 4.1.8 Pachymic acid

Pachymic acid (Pac) is a metabolite derived from *Wolfiporia cocos* (F. A. Wolf) Ryvarden & Gilb. Pac supplementation alleviated hepatic inflammation in HFD-induced NAFLD mouse models (Ren et al., 2025a). Pac reshaped the GM structure in NAFLD mice, reversing HFD-induced intestinal dysbiosis. At the phylum level, the *Firmicutes*/*Bacteroidetes* ratio decreased. At the genus level, the abundance of *Akkermansia* increased, while *Desulfovibrio* and *Streptococcus* decreased. Furthermore, Pac reduced the expression of hepatic inflammatory factors by suppressing the LPS/TLR4/MYD88/NFκB pathway, thereby mitigating liver inflammation. Pac inhibits the expression of lipid synthesis-related proteins including FASN, SREBP1c, and SCD1, while promoting the expression of fatty acid oxidation-related proteins such as PPARα and CPT1α, thereby reducing hepatic inflammation induced by lipid accumulation. Studies demonstrate that Pac ameliorates HFD-induced NAFLD through synergistic multi-pathway effects. In animal models, Pac supplementation (20/40 mg/kg for 4 weeks) significantly alleviates hepatic steatosis, reduces serum lipid levels, and improves liver function (Ren et al., 2025b). On one hand, Pac upregulates the expression and activity of PPARα, thereby promoting hepatic fatty acid oxidation to accelerate lipid consumption. Additionally, activated PPARα upregulates GPX4 protein expression, thus inhibiting ferroptosis. On the other hand, Pac downregulates TFR1 protein expression by inhibiting the MAPKs signaling pathway, consequently reducing Fe^3+^ uptake and intracellular Fe^2+^ accumulation in hepatocytes. This suppresses the Fenton reaction and further alleviates hepatocellular ferroptosis. Through multiple mechanisms including promoting lipid metabolism, inhibiting inflammation, and suppressing ferroptosis, Pac collectively delays the progression of NAFLD.

### 4.2 Botanical drugs formulae

Botanical drugs formulae comprise complex systems of multiple botanical drugs, corresponding to diverse metabolites. Their core therapeutic mechanism lies in the synergistic effects of metabolites through multi-target and multi-system interventions, achieving holistic regulation of diseases.

Zexie Tang contains two botanical drugs: *Alisma plantago-aquatica* L. and *Atractylodes macrocephala* Koidz. Researchers discovered that Zexie Tang contains a metabolite composed of fructose and glucose, designated as Zexie Tang Polysaccharides (ZXTPs) (Zhang et al., 2025). ZXTPs ameliorate hepatic steatosis in NAFLD mouse models via the gut-liver axis. ZXTPs remodeled the GM, increasing the abundance of beneficial bacteria including *Akkermansia*, *Lachnospiraceae_NK4A136*, and *Bacteroides*, while reducing pathogenic bacteria such as *Prevotella_9* and *Phascolarctobacterium*. This alteration promoted the secretion of tryptophan metabolites (including indole-3-acetic acid and serotonin) and SCFAs. Tryptophan metabolites play a key role in alleviating hepatic inflammation and modulating immunity by activating AhR. Concurrently, ZXTPs upregulate the expression of tight junction proteins (ZO-1 and Occludin), repairing the intestinal mucosal barrier and thereby inhibiting gut-derived LPS from entering systemic circulation. Meanwhile, tryptophan metabolites exert crucial effects in mitigating hepatic inflammation and modulating immunity through activation of the AhR. Regarding lipid metabolism, SCFAs and ZXTPs enhance hepatic AMPK phosphorylation through regulating LKB1/AMPK and PI3K/AKT/mTOR signaling pathways along with the autophagy pathway. This subsequently suppresses expression of the lipogenesis key enzyme SREBP1 while upregulating PPARα expression, ultimately leading to significant amelioration of hepatic lipid metabolism.

Yinzhihuang granule (YZHG) contains four types of botanical drugs, including *Artemisia capillaris* Thunb, *Gardenia jasminoides* J.Ellis, *S. baicalensis* Georgi, *Lonicera japonica* Thunb. The study (Tan et al., 2023) revealed that YZHG contains 42 blood-absorbed metabolites, primarily flavonoids, phenolic acids, iridoids. YZHG could reduce blood lipid levels in NAFLD mouse models and decrease concentrations of LPS, TNF-α, IL-1β, and IL-6 in liver tissues. YZHG modulated the GM structure in mice: at the phylum level, the abundance of *Firmicutes* and *Proteobacteria* decreased, while *Patescibacteria* and *Tenericutes* increased; At the genus level, the abundance of *Ruminococcaceae_UCG-014*, *Lactobacillus*, and *Desulfovibrio* elevated. 16S rRNA sequencing and metabolomics demonstrated that this therapeutic effect stems from YZHG metabolites influencing intestinal tract and lipid metabolism-related proteins, particularly chrysin, baicalein, wogonin, hispidulin, and negletein A.

Xie Zhuo Tiao Zhi formula (XZTZ) contains *Crataegus pinnatifida* Bunge, *Nelumbo nucifera* Gaertn., *Citrus × aurantium* Siebold & Zucc. ex Engl., *A. macrocephala* Koidz., *W. cocos* (F. A. Wolf) Ryvarden & Gilb, *A. plantago-aquatica* L. These botanical drug-derived metabolites (including naringin, neohesperidin, atractylenolide III, 23-acetyl alisol B, pachymic acid, and ursolic acid) elevate circulating and hepatic inosine levels by increasing the abundance of *A. muciniphila*, *Bifidobacterium pseudolongum*, and *Ileibacterium valens* in the intestinal tract of NAFLD mouse models. This subsequently inhibits hepatocyte pyroptosis, as evidenced by downregulation of NLRP3, GSDMD, Nek7, Caspase-1, and ASC protein expression, while reducing inflammatory factors such as IL-1β, IL-6, and TNF-α. Concurrently, other metabolites (such as Inosine) effectively alleviate hepatic lipid accumulation by regulating the expression of proteins involved in lipid synthesis, transport, and oxidation (Qiu et al., 2023).

Yinchen-Gancao decoction (YG) consists of two botanical drugs: *A. capillaris* Thunb. and *Glycyrrhiza uralensis* Fisch., which carry multiple metabolites, including Chlorogenic Acid (CGA), Glycyrrhizic Acid (GZA), Isochlorogenic Acid (ICGA), and glycyrrhetinic acid (GTA). YG significantly ameliorates hepatic lipid accumulation and inflammation in the NASH mouse model. This effect stems from CGA suppressing the fatty acid synthesis pathway (SREBP1c-ACC/FASN) via an FXR-dependent mechanism, downregulating the expression of the uptake protein CD36, and enhancing lipid oxidation through the PPARα-CPT1α pathway (Jing et al., 2025).

Si-Wu-Tang (SWT) treats CCL4-induced hepatic fibrosis by remodeling the composition of GM and regulating BAs metabolism (Xue et al., 2021). Twenty-two major metabolites derived from botanical drugs in SWT, particularly paeoniflorin, ferulic acid, verbascoside, and senkyunolide A, play crucial roles. They regulate BAs metabolism by activating the FXR-fibroblast growth factor 15 (FGF15) and FXR-SHP pathways, which facilitates hepatic lipid excretion and reduces lipotoxicity-induced inflammation. Conversely, paeoniflorin and ferulic acid synergistically improve the intestinal microenvironment, at the phylum level, the abundance of *Bacteroides* and *Lachnoclostridium* increases; at the genus level, the abundance of *Alistipes* decreases. At the family level, reduced abundance of *Rikenellaceae* suppresses gut-derived endotoxin translocation, thereby alleviating hepatic inflammation burden.

Lingguizhugan decoction (LGZG) can alleviate hepatic inflammation in HFD-fed mice. This effect is closely associated with metabolites derived from botanical drugs, including Paclitaxel (Pac), Cinnamaldehyde, Atractylenolide II, and Glycyrrhizic acid (GZA). These metabolites inhibit TNFα and IFNβ release in a dose-dependent manner; Notably, both Cinnamaldehyde and GZA further block activation of the STING–TBK1–NF-κB signaling pathway by suppressing TBK1 and NF-κB phosphorylation in macrophages. Notably, the effects of mixed metabolites surpassed those of single metabolites, indicating synergistic interactions among metabolites (Cao et al., 2022). Yindanxinnaotong (YDX) comprises eight botanical drugs containing a total of 124 metabolites. Studies demonstrate that YDX reduces gut-derived LPS production by remodeling the GM. At the genus level, it significantly increases the abundance of *Odoribacter*, *Alistipes*, and *Flavonifractor* while decreasing *Clostridium cluster XIVa* and *Barmesiella*. Furthermore, YDX downregulates hepatic expression of lipid synthesis-related proteins (including SREBP-1c, SCD-1, and CD36) and pro-inflammatory cytokines (IL-6, TNF-α), while enhancing expression of key fatty acid β-oxidation proteins (AMPKα, CPT-1). This effectively suppresses hepatic lipid accumulation and inflammatory responses (Huang et al., 2024). Although studies have observed alterations in GM alongside improvements in hepatic lipid metabolism and inflammatory status, the direct link between these two pathways, the primary metabolites involved, and the specific mechanisms remain to be elucidated, this warrants further investigation.

Shugan Xiaozhi (SG), composed of 15 botanical drugs, is commonly employed in the treatment of NAFLD. SG ameliorates hepatic inflammation and fibrosis in HFD-induced mice, suppresses intrahepatic ROS generation, elevates levels of SOD, GSH, and CAT, reduces MDA levels in murine liver in a dose-dependent manner, and preserves the integrity of hepatic mitochondrial function and structure. These effects depend on SG’s regulation of BNIP3/BNIP3L-mediated mitophagy. Metabolites derived from SG—including naringin, hesperetin 7-O-rutinoside, frangulin A, and 3″-p-Coumaroylprunin—exhibit close interactions with key targets regulating mitophagy, suggesting their pivotal roles in this process (Chen M. et al., 2023).

Shugan Xiaozhi (SG), composed of 15 botanical drugs, is commonly used to treat NAFLD. Studies demonstrate that SG alleviates HFD-induced hepatic inflammation and fibrosis in mice, suppresses ROS generation, enhances SOD, GSH, and CAT activities, and reduces MDA levels in a dose-dependent manner, thereby protecting mitochondrial structural and functional integrity in hepatocytes. The therapeutic effect of SG on NASH is closely associated with its regulation of BNIP3/BNIP3L-mediated mitophagy. Metabolites in SG—including naringin, hesperetin 7-O-rutinoside, frangulin A, and 3″-p-Coumaroylprunin—exhibit significant interactions with key mitophagy targets, thereby playing a central role in this mechanism (Chen M. et al., 2023).

## 5 Discussion

**FIGURE 4:**
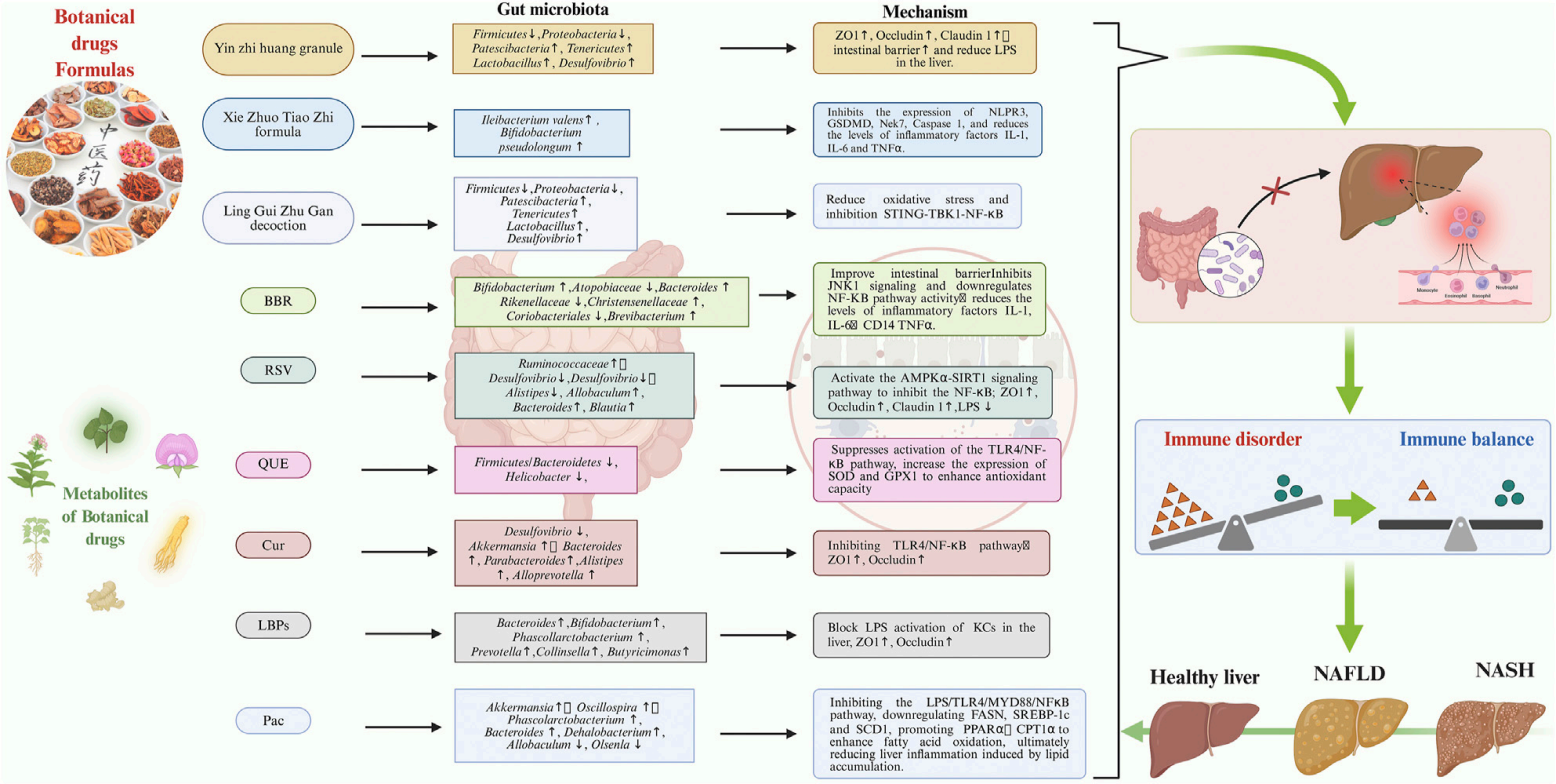
The specific targets and mechanisms of action of botanical drugs formulae and metabolites from botanical drugs, in treating NAFLD by targeting the GM-immune response. GM, gut microbiota; NAFLD, Non-alcoholic Fatty Liver Disease.

Immune dysregulation serves as a critical driver of NAFLD progression, making the maintenance of immune homeostasis an essential intervention strategy. GM regulates host immune balance through the gut-liver axis and participates in hepatic inflammation processes. Therefore, utilizing metabolites derived from botanical drugs to treat NAFLD through the GM-Immune Axis represents a promising strategy. As shown in Figure 4. However, its mechanisms remain incompletely elucidated and face multifaceted challenges.

GM is highly susceptible to environmental influences, resulting in significant research heterogeneity. This necessitates integrating multi-center large-sample cohorts with multi-omics data to enhance conclusion reliability. Organoid co-culture systems (Wang et al., 2024a) and CRISPR-based microbiota editing (Jin et al., 2022) provide novel approaches to overcome mechanistic bottlenecks. The former can simulate gut-liver axis immune interactions for target screening, while the latter enables precise identification of functional genes in GM and drug-action pathways.

The clinical efficacy evidence and translation of botanical drugs still face dilemmas. Current research predominantly focuses on basic mechanisms, with limited and low-quality clinical studies. These manifest experimental design flaws (e.g., inadequate sample size, suboptimal blinding, insufficient endpoint indicators, significant regional variations, and short follow-up periods), lax quality control, and lack of collaborative mechanisms, substantially diminishing the evidence level. Future efforts should initiate pilot experiments and observational studies to preliminarily evaluate efficacy, identify benefiting subpopulations, and define treatment endpoints, thereby providing foundations for subsequent large-scale RCTs. Studies must strictly adhere to the PICOTS framework, establish intelligent data centers, and follow international reporting standards (e.g., CONSORT, STRICTA) to advance the generation of high-quality clinical evidence.

The translation from basic research to clinical applications of botanical drugs also faces challenges. The multi-target characteristics result in unclear onset of action metabolites and dose-response relationships, while the lack of standardized preparation processes leads to inconsistent drug quality and irreproducible efficacy (Hu et al., 2019). Multidisciplinary platforms should be integrated to establish artificial intelligence-based metabolites screening and quality control systems, evaluate pharmacological effects using organoids/organs-on-chips, and ultimately develop a research paradigm featuring well-defined mechanisms, rigorous quality control, and quantifiable efficacy.

The safety of metabolites derived from botanical drugs also requires significant attention. Toxic side effects may be associated with exogenous contaminants (pesticides, heavy metals, mycotoxins), endogenous factors (origin, dosage, treatment duration, preparation processes), and individual variations (Gao et al., 2019). Current toxicological research remains limited, with unclear toxicity mechanisms and a lack of clinical risk warnings. Regulatory oversight of drug quality should be enhanced, toxicity evaluation systems improved, toxicity metabolites pre-screened using chemical structure warning databases, pharmacokinetic-toxicokinetic (PK-TK) models established through integrated *in vivo* and *in vitro* experiments, and novel technologies such as microfluidic chips, high-throughput screening, and systems toxicology introduced to develop more comprehensive safety evaluation standards.

In summary, elucidating the interaction targets among botanical drugs, GM, and immune dysregulation provides novel insights for NAFLD treatment, deepening our understanding of the gut-liver axis mechanism. Integrating mechanistic studies, clinical validation, and safety assessment holds promise for advancing systematic, standardized, and internationally recognized applications of botanical drugs in NAFLD treatment.
